# Green Synthesis of ZnSe Nanoparticles via Laser Fragmentation: Effect of Laser Pulse Energy on Nanoparticle Size and Surface Phonon Modes

**DOI:** 10.3390/nano16030206

**Published:** 2026-02-05

**Authors:** Patricia Maldonado-Altamirano, Maria de los Angeles Hernandez-Perez, Luis Arturo Martínez-Ara, Jorge Sastré-Hernández, Jaime Santoyo-Salazar

**Affiliations:** 1 Escuela Superior de Física y Matemáticas, Instituto Politécnico Nacional, Edificio 9, U.P.A.L.M., San Pedro Zacatenco, Ciudad de México 07738, Mexico; mtzara1984@gmail.com (L.A.M.-A.); jsastre@ipn.mx (J.S.-H.); 2 Instituto Politécnico Nacional, Departamento de Ingeniería en Metalurgia y Materiales, Escuela Superior de Ingeniería Química e Industrias Extractivas, U.P.A.L.M., San Pedro Zacatenco, Ciudad de México 07738, Mexico; 3 Centro de Análisis y Pronósticos Meteorológicos Aeronáuticos, Servicios a la Navegación en el Espacio Aéreo Mexicano. Av. 602 161 Zona Federal, Aeropuerto Internacional de la Ciudad de México, Ciudad de México 15620, Mexico; 4 Departamento de Física, Centro de Investigación y de Estudios Avanzados del Instituto Politécnico Nacional, Av. Instituto Politécnico Nacional 2508, San Pedro Zacatenco, Gustavo A. Madero, Ciudad de México 07360, Mexico; jaime.santoyo@cinvestav.mx

**Keywords:** ZnSe nanoparticles, UV-Vis, Raman, Surface mode

## Abstract

ZnSe nanoparticles were synthesized via the sustainable laser fragmentation in liquids (LFL) technique using a Nd:YAG laser at 1064 nm. The pulse energy was varied to study its effect on the particle size and vibrational properties. UV–Vis absorption spectra show a blue shift in the absorption edge with a decreasing pulse energy. The sample processed at the lowest pulse energy has the smallest nanoparticles (10.3 nm average), reaches an optical band gap of 2.83 eV, and exhibits a high-energy shoulder attributed to spin–orbit-related transitions. Raman spectra reveal a strong enhancement of the surface phonon mode (231–234 cm^−1^), where its intensity surpasses that of the longitudinal optical mode, demonstrating the dominant role of surface atoms in the vibrational response. TEM confirms a wide size distribution, i.e., centered at 10.3 ± 6.4 nm, which can account for the simultaneous observation of bulk-like and quantum-confined optical and Raman features. These results show that the pulse energy effectively tunes the nanoparticle size and phonon behavior, positioning LFL as a clean and versatile method for producing ZnSe nanostructures with relevant properties for optoelectronic applications.

## 1. Introduction

Zinc selenide (ZnSe) is a II–VI direct band gap semiconductor (Eg ≈ 2.7 eV for the bulk crystal) that has attracted extensive attention for optoelectronic applications, including light-emitting diodes, photodetectors, and color display technologies. ZnSe nanoparticles can also be functionalized for biomedical applications such as cancer diagnostics and drug delivery due to their relatively low toxicity [1,2], as well as for photocatalytic processes [3,4]. ZnSe typically crystallizes in two main structures: the cubic zinc-blende and the hexagonal wurtzite structures. Reports of wurtzite-phase ZnSe nanoparticles are less common, as this phase is metastable under ambient conditions and typically requires specific synthesis routes to form and persist [5,6].

In recent years, ZnSe nanoparticles have gained interest as platforms for solar energy conversion, photocatalysis, optical devices, and health-care technologies due to their tunable band gap, high photochemical stability, low toxicity, and cost-effectiveness [3]. As the particle size approaches the exciton Bohr radius, quantum confinement effects emerge, modifying electronic and vibrational properties and enabling size-dependent optical responses.

Laser fragmentation in liquids (LFL) offers significant advantages for the synthesis of high-purity, crystalline nanoparticles. Conventional II–VI semiconductor synthesis methods, such as colloidal, hot-injection, solvothermal or hydrothermal, microemulsion, and mechanochemical techniques, typically depend on molecular precursors, surfactants, capping agents, or organic solvents to control nucleation and growth. Nanoparticles produced by these approaches often retain surface-bound residues, which can negatively affect optical properties, chemical stability, or biocompatibility. In contrast, LFL is a surfactant- and additive-free physical process that does not require external reagents or generate by-products, resulting in chemically pure colloidal nanoparticles. This method is particularly advantageous when surface functionality and purity are essential, as in optoelectronics, catalysis, or biomedical interface engineering. Furthermore, LFL aligns with green chemistry principles by minimizing waste, improving energy efficiency, and eliminating hazardous substances, thereby offering a sustainable and scalable approach to nanomaterial synthesis [7].

This top-down physical method uses pulsed laser radiation to irradiate a microcrystalline suspension of the target material. The laser triggers particle breakdown through thermal, non-thermal, or mechanical mechanisms, depending on the laser parameters and the system’s optical properties. In thermal processes, the material absorbs laser photons and heats rapidly, reaching extreme temperatures near the critical point, which results in the melting and evaporation of the particle. Mechanical fragmentation is associated with the expansion and collapse of a cavitation bubble formed near the irradiated region. The violent collapse of the bubble produces shockwaves and pressure transients that mechanically remove surface layers from the particle, particularly where the expansion meets resistance from the surrounding liquid. Non-thermal processes, commonly induced by ultrashort femtosecond pulses, involve rapid ionization prior to significant thermal coupling with the crystal lattice. This causes the ejection of free electrons and the accumulation of a positive charge within the nanoparticle, resulting in a strong Coulombic repulsion that destabilizes and disintegrates the particle. These mechanisms determine the degree of fragmentation, particle size distribution, and crystallinity [8,9,10,11,12]. LFL enables control over the particle size and crystallinity by adjusting the laser parameters, like the pulse duration and repetition (not easily modified), fluence, and wavelength, making it a versatile tool for nanomaterial engineering.

LFL has been successfully employed to synthesize size-tunable, ligand-free nanoparticles of II–VI semiconductors, which exhibit well-defined optical and structural properties. Although CdSe quantum dots with diameters of 2–8 nm have been extensively investigated and show clear signatures of quantum confinement in their absorption and emission spectra [9,11], reports on ZnSe nanocrystals remain limited despite their potential for similar applications. Nonetheless, recent studies indicate that ZnSe nanoparticles synthesized by LFL exhibit phase stabilization effects, strong surface-related emissions, and Raman signals that change via nanoscale size dispersion [13].

In this work, we investigate the influence of pulse energy on the synthesis of ZnSe nanoparticles via LFL. Special attention is given to the relationship between the particle size and vibrational dynamics, with an emphasis on the enhancement of the surface phonon mode relative to the longitudinal optical (LO) phonon. Understanding this correlation contributes to the broader effort to tailor the optical and vibrational responses of ZnSe nanoparticles for future optoelectronic and photonic applications.

## 2. Materials and Methods

### 2.1. Synthesis of ZnSe Nanoparticles

ZnSe nanoparticles were synthesized using the laser fragmentation in liquids (LFL) technique with a Q-switched Nd:YAG laser Spectra-Physics, Quanta-Ray (Milpitas, CA, USA) operating at 1064 nm, with a pulse duration of 12 ns and a repetition rate of 50 Hz. The laser pulse energy varied between 4.4 and 24 mJ to investigate its effect on nanoparticle size.

**Figure 1:**
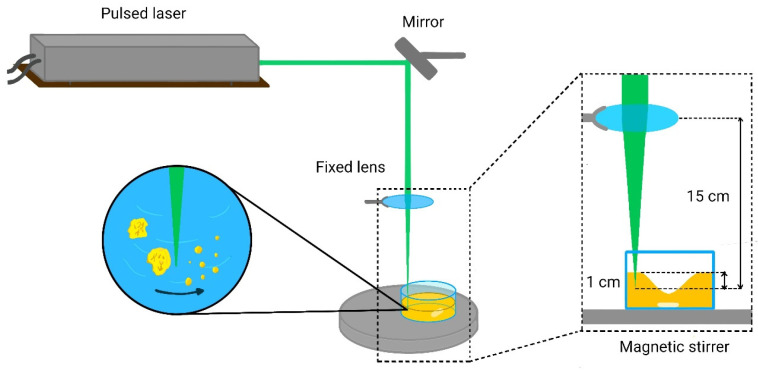
A schematic illustration of the experimental setup used for the synthesis of ZnSe nanoparticles via LFL.

Four samples were prepared at varying laser pulse energies, as detailed in Table 1. These conditions were chosen based on prior studies on the LFL of CdSe [9]. For each experiment, 0.03 g of ZnSe microcrystalline powder (Sigma-Aldrich, St. Louis, MO, USA, 99.99%) was dispersed in 40 mL of acetone within a Pyrex crystallizer. Acetone served as the liquid medium due to its relatively inert properties during laser fragmentation, which limit surface oxidation of ZnSe nanoparticles and promote colloidal stability after synthesis [14]. The suspension was magnetically stirred at 400 RPM throughout the 30 min irradiation to maintain homogeneity. The laser beam was focused approximately 1 cm below the liquid–air interface using a mirror–lens assembly, as schematically shown in Figure 1. The focal point position was selected by considering the acetone volume, crystallizer dimensions, and the suspension vortex. To maintain a constant acetone volume, the suspension height was calibrated during stirring and adjusted by adding acetone dropwise during irradiation. The total acetone volume was maintained at 40 ± 4.24 mL for all experiments.

After LFL, the acetone volume was adjusted to 40 ± 1.4 mL. The crystallizer was then sealed and left undisturbed. On the first, second, and fifth days after laser ablation, the supernatant was decanted to separate it from the solids, with the supernatant volume consistently maintained at 40 ± 1.4 mL. No additional precipitation or turbidity was observed after the final decantation. The samples were subsequently stored in sealed vials until characterization.

### 2.2. Characterization

For structural analysis, 1 mL of nanoparticle suspensions were drop cast onto Corning glass substrates and dried under room conditions. The dried samples were analyzed using a Bruker D8 Advance diffractometer (Billerica, MA, USA) with Cu Kα radiation (λ = 1.5406 Å) in grazing incidence geometry operating at 0.3°, 0.02°/step, and 10 s/step.

Morphological analysis was performed using transmission electron microscopy (TEM) with a JEOL JEM-2010 microscope (Tokyo, Japan) operating at 200 kV. Suspensions were deposited onto 400-mesh Formvar/carbon-coated copper grids and allowed to dry at room temperature.

UV–Vis absorption spectra were recorded using a Perkin Elmer Lambda 35 (Waltham, MA, USA) double-beam spectrophotometer. Measurements were performed in Brand UV cuvettes with a 1 cm path length. The acetone background spectrum was subtracted from each measurement to isolate the optical contribution of the nanoparticles. Cuvettes were kept closed during scan to prevent acetone evaporation.

The drop-cast films prepared for XRD analysis were also used for Raman measurements. Raman spectra were acquired using a Horiba Jobin Yvon LabRAM HR Evolution (Longjumeau, France) spectrometer with excitation provided by a 633 nm He–Ne laser. The laser beam was focused with a 100× objective, and a 25% optical density filter was used to prevent local heating. Spectra were recorded at room temperature under dark conditions. To enhance the signal-to-noise ratio and ensure sample representativeness, three spectra were acquired at distinct positions for each sample and subsequently averaged.

## 3. Results and Discussion

All ZnSe samples exhibited a yellow, turbid appearance immediately after synthesis, consistent with the presence of micro- and nanocrystalline ZnSe. The turbidity is attributed to light scattering from residual microcrystals that were not fully fragmented during irradiation. After the decantation process, the supernatant visual intensity of the yellow coloration varied among the samples: the sample synthesized at the lowest pulse energy exhibited the lightest yellow color, while the sample synthesized at the highest pulse energy showed the most intense yellow coloration. These variations are attributed to changes in the nanoparticle concentration and the resulting particle size distribution. Importantly, the supernatant nanoparticle dispersions remained optically stable for several months without visible aggregation or sedimentation.

### 3.1. XRD

**Figure 2:**
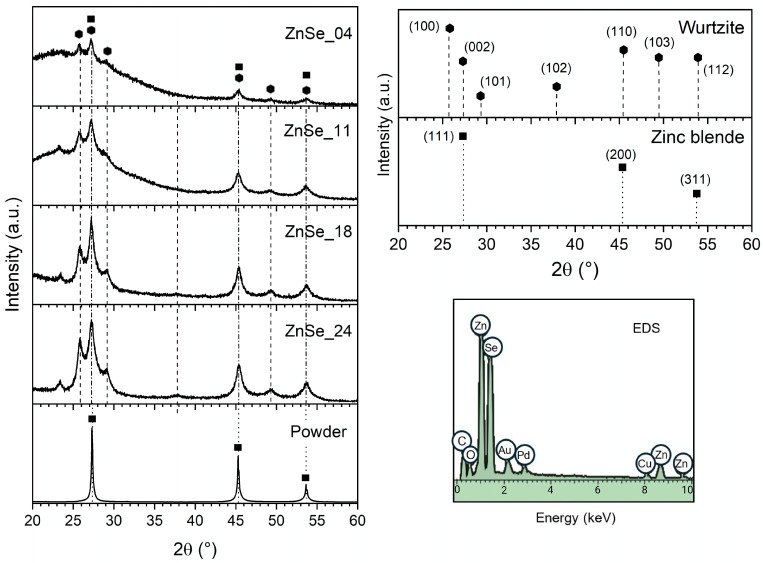
X-ray diffraction patterns of ZnSe nanoparticle samples, precursor powder, and reference patterns for zinc-blende and wurtzite structures and representative EDS spectrum.

Figure 2 shows the X-ray diffraction patterns of the ZnSe nanoparticle samples and the precursor powder. The reference patterns of the zinc-blende and wurtzite structures are also included. The precursor exhibits the characteristic reflections of cubic zinc-blende ZnSe at 2θ = 27.3°, 45.3°, and 53.6°, corresponding to the (111), (220), and (311) planes, according to JCPDS card No. 37-1463 [15,16]. These reflections are indicated by ◼ and highlighted by dotted lines.

In contrast, the nanoparticle samples display several additional reflections absent in the zinc-blende structure. Peaks located at 2θ ≈ 25.7°, 27.2°, 29.1°, 37.8°, 45.3°, 49.3°, and 53.6° can be indexed to the (100), (002), (101), (102), (110), (103), and (112) planes of the wurtzite ZnSe (JCPDS 80-0008). These peaks are labeled by ⬢ and marked with dashed lines. Their presence provides direct evidence of hexagonal stacking sequences and indicates that laser fragmentation stabilizes wurtzite-like motifs, as previously reported for ZnSe nanoparticles synthesized via femtosecond laser ablation [5]. However, a zinc-blende structure has been also reported for ZnSe nanoparticles processed using laser ablation in deionized water [17]. Given the well-known zinc-blende/wurtzite polytypism of II-VI and III-V semiconductors, cubic and hexagonal domains may coexist in samples. Subtle differences between these structures—like layer stacking and dihedral conformation, ABCABC and staggered for the zinc-blende and ABABAB and eclipsed for the wurtzite—lead some interplanar distances to be equivalent. As a result, the correspondent XRD peaks overlap [18]. In Figure 2, the overlaps are indicated by a dash–dot line to ▪(111)-⬢(002), ▪(220)-⬢(110), and ▪(311)-⬢(112) pairs of planes.

The overall intensity of the diffraction peaks is markedly lower in the nanoparticle samples than in the precursor powder. This behavior is attributed to the smaller amount of diffracting material and to the increased structural disorder in the drop-casted nanoparticle films, rather than to a simple thickness effect. Given the limited peak definition, the grazing incidence geometry, and the presence of an amorphous band, no attempt was made to extract crystallite sizes from line broadening; instead, the nanoparticles’ size distribution was quantified by TEM, as discussed in Section 3.2 [19]. The amorphous band could be associated with the quartz substrate; however, it is more pronounced in samples processed at a low pulse energy, which may be related to their lower nanoparticle density combined with partial amorphization effects in smaller nanoparticles.

A weak feature near 23.4° is observed in all nanoparticle samples, whose intensity increases with the synthesis pulse energy. This reflection does not correspond to any ZnSe plane from either the cubic or hexagonal phases. Similar low-intensity peaks have been reported in ZnSe nanostructures synthesized under energetic or laser-based conditions and have been attributed to surface carbon formed by the interaction of acetone surrounding the hot ablated matter [8,9,14]. A low-intensity peak around 24° has been observed and attributed to ZnSeO_4_ produced during ZnSe powder laser ablation [17] and the chemical synthesis of ZnSe quantum dots [20]. Figure 2 shows a representative energy-dispersive X-ray spectroscopy (EDS) spectrum of ZnSe nanoparticles. The presence of Zn and Se signals confirms the nanoparticle formation. Cu, Au, and Pd signals come from the Cu tape and Au-Pd coating employed to enhance conductivity. The peaks corresponding to C and O are likely associated with the aforementioned C and ZnSeO_4_ species.

### 3.2. TEM

**Figure 3:**
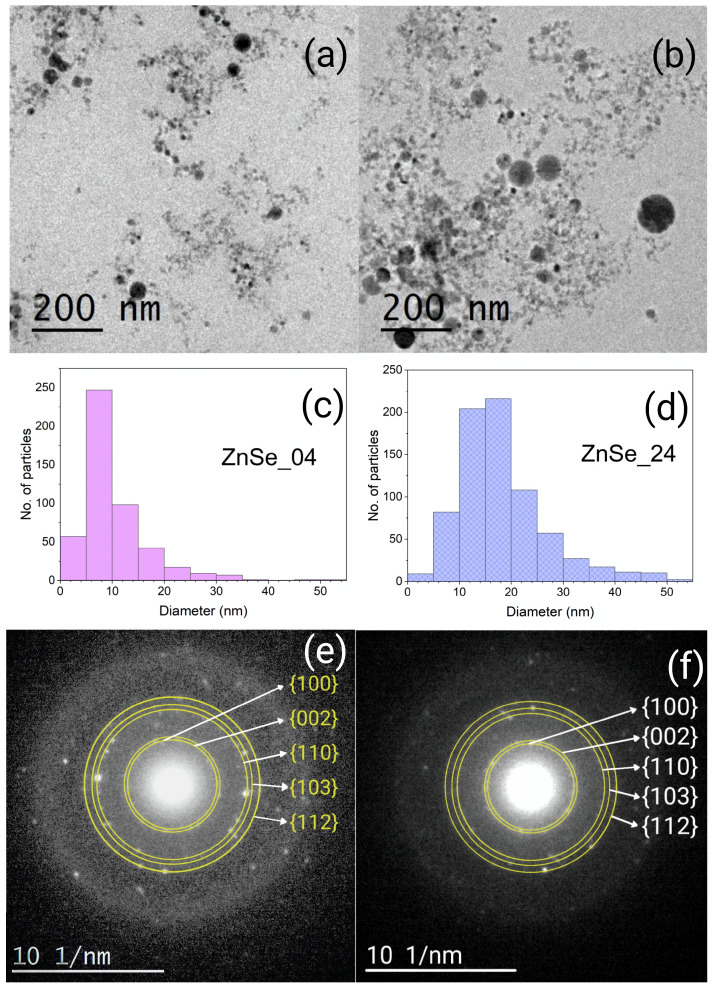
(**a**,**b**) Low-magnification TEM images, (**c**,**d**) particle size distribution histograms, and (**e**,**f**) SAED patterns of ZnSe nanoparticles synthesized at 4.4 mJ/pulse and 24 mJ/pulse. In the SAED patterns, the families of crystallographic planes are presented in {}.

Figure 3a,b show representative TEM micrographs of the ZnSe nanoparticles synthesized at the lowest (ZnSe_04) and highest (ZnSe_24) pulse energies, respectively. Both images were acquired at the same magnification (200 nm scale), allowing a direct comparison of their morphologies. The ZnSe_04 sample exhibits a large population of small, well-dispersed nanoparticles, whereas the ZnSe_24 sample displays a markedly broader range of particle sizes together with several large fragments that were not fully broken during irradiation.

The particle size histograms presented in Figure 3c,d are derived from the analysis of seven transmission electron microscopy (TEM) micrographs per sample. These micrographs were obtained at magnifications ranging from 20 to 200 nm, providing representative statistics across the entire particle size range. For the ZnSe_04 sample, 480 nanoparticles were measured, while 748 nanoparticles were analyzed for ZnSe_24. To improve the clarity of the statistical analysis, a small number of large, rare particles were excluded from histograms. In the ZnSe_04 sample, four particles with diameters between 75 and 85 nm were omitted. In the ZnSe_24 sample, twelve particles were excluded: two with diameters of 178 and 109 nm and ten with diameters between 60 and 75 nm. These excluded particles remain visible in the TEM micrographs.

The ZnSe_04 sample displays a relatively narrow particle size distribution centered at 8–10 nm. The average diameter is 10.3 nm, with a standard deviation of 6.4 nm. Notably, about 6.6% of the particles (32 particles) have diameters below 4 nm, which are comparable to or smaller than the ZnSe exciton Bohr radius (~4 nm). In contrast, the ZnSe_24 sample exhibits a broader particle size distribution, with an average diameter of 18.4 nm and a standard deviation of 9.0 nm. In this case, only 0.8% of the particles (6 particles) have diameters below 4 nm, indicating a significantly reduced contribution from strongly confined nanoparticles.

The observed evolution of the particle size distribution with pulse energy suggests a change in the fragmentation regime during LFL. At lower pulse energies, fragmentation proceeds in a comparatively mild manner, favoring the formation of smaller and more uniform nanoparticles. In contrast, increasing the pulse energy leads to a more disruptive breakup process, resulting in a broader size distribution, with small nanoparticles coexisting with larger fragments that are not fully fragmented during irradiation [14,21].

Figure 3e,f show the selected area electron diffraction (SAED) patterns of the ZnSe_04 and ZnSe_24 samples, respectively. Both exhibit concentric diffraction rings, indicating that the nanoparticles are polycrystalline. The rings have been highlighted to facilitate identification. The rings in the ZnSe_04 sample are sharper, consistent with its narrower particle size distribution, whereas the ZnSe_24 sample shows more diffuse rings, reflecting its broader size range and local strain variations. The measurement of the ring spacings (Table 2) matches well with the reference d-values of wurtzite ZnSe (JCPDS 80-0008), in agreement with the wurtzite-like reflections observed in XRD. In particular, the first three rings correspond to the families of planes {002}, {101}, and {110}, providing direct evidence of hexagonal stacking sequences in the nanoparticles. The small deviations between the experimental d and reference d (typically <5%) can be attributed to strain and size effects commonly observed in II–VI nanocrystals or to the coexistence of cubic and hexagonal stacking in the nanoparticles, consistent with the polytypism reported for ZnSe nanostructures.

Overall, TEM and SAED analyses demonstrate that the pulse energy strongly influences the particle size and local crystallinity. Low-power irradiation produces smaller and more uniform nanoparticles, while high-power irradiation yields a mixture of small particles and larger fragments. These trends directly correlate with the optical and vibrational behavior discussed in the following sections.

### 3.3. UV-Vis

**Figure 4:**
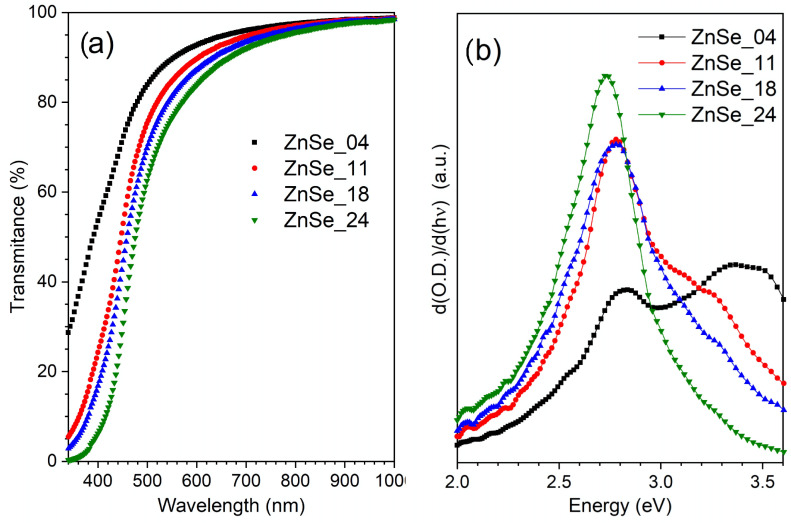
(**a**) Optical transmittance spectra and (**b**) the first derivative of the optical density of the samples.

Figure 4a shows the transmittance spectra of the ZnSe nanoparticle dispersions synthesized at different pulse energies. The spectra were not normalized.

No scattering from microcrystals is evident above 700 cm^−1^, indicating the absence of microcrystals. The sample obtained at the highest pulse energy (ZnSe_24) exhibits a relatively abrupt absorption edge near the expected region for bulk-like ZnSe. As the laser power decreases (ZnSe_18, ZnSe_11, and ZnSe_04), the absorption edge progressively shifts to a lower wavelength. The most pronounced shift is observed for ZnSe_04, which does not reach zero transmittance within the measured spectral window. This behavior is consistent with the presence of smaller nanoparticles and an increased size dispersion, as evidenced by the TEM analysis [22,23,24].

To extract the optical band gap, the optical density was calculated from the transmittance spectra, and its first derivative with respect to photon energy was evaluated, the results are presented in Figure 4b. The derivative method enhances subtle variations near the absorption edge and allows for the identification of the energy corresponding to the most probable electronic transition [25,26].

As shown in Figure 4b and Table 1, the ZnSe_24 sample exhibits a well-defined maximum at 2.71 eV, a value close to the bulk ZnSe. As the synthesis power decreases, this maximum shifts to higher energies, yielding band gap values of 2.77 eV (ZnSe_18 and ZnSe_11) and 2.83 eV for the lowest-power sample, ZnSe_04. The progressive blue shift correlates directly with the reduction in the particle size observed via TEM, reflecting the onset of quantum confinement effects in the smallest nanoparticles of the distribution [27,28].

In addition to the main derivative peak, a secondary feature appears at higher energies: a shoulder for the ZnSe_24 evolves to a clear band for ZnSe_04. We attribute this signal to transitions involving the spin–orbit split-off valence band. Although the exact energy separation between valence band sublevels depends on the crystal structure, strain, and confinement, several recent studies on ZnSe nanocrystals report that SO-related transitions can become more prominent when nanoscale effects, surface states, and phase mixing (wurtzite/zinc-blende polytypism) perturb the valence band structure [29,30]. Thus, the presence and amplification of this high-energy shoulder in the smallest nanoparticles is consistent with expected band structure modifications in low-dimensional II–VI semiconductors.

It is important to note that, although the average particle sizes determined by TEM are larger than the exciton Bohr radius of ZnSe (3.8 nm) [31], both the broad size distribution and the long tail toward sub 5 nm diameters suggest that a fraction of the nanoparticles may indeed fall within the strong confinement regime. This fraction, though small, is sufficient to enhance SO-related transitions in the derivative spectra, in agreement with the behavior reported for size-dispersed ZnSe quantum dots.

Overall, the UV–Vis results reveal a clear dependence of the optical band gap and higher-energy transitions on the pulse energy used during synthesis, reflecting the strong influence of the nanoparticle size and structural heterogeneity on the electronic structure of ZnSe nanocrystals.

### 3.4. Raman

Raman spectra were recorded at room temperature under dark conditions. For each sample, the reported spectrum represents the average of at least three analyzed regions. A baseline correction was performed using the asymmetric least squares (AsLS) method within the 110–390 cm^−1^ spectral range, employing an asymmetry factor of 0.001. The analyses were conducted on the baseline-subtracted spectra.

**Figure 5:**
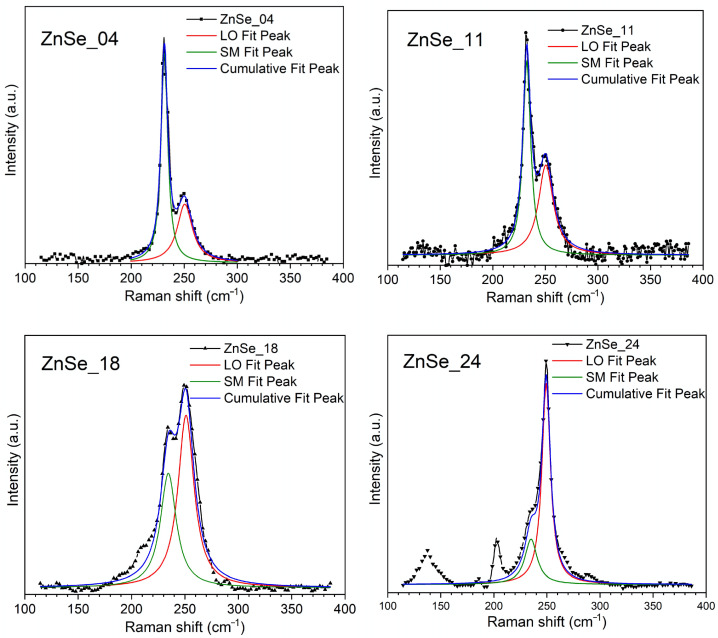
Raman spectra and Lorentzian fitting curves for the SM and LO modes of ZnSe nanoparticles synthesized at different pulse energies.

Figure 5 shows the Raman spectra of the four ZnSe nanoparticle samples; all spectra display a strong band at 250 cm^−1^, which is assigned to the longitudinal optical (LO) phonon mode of ZnSe [32,33]. In addition, a second band located at ~231–234 cm^−1^ is observed, which is attributed to the surface phonon mode (SM) [34]. The SM appears when the particle dimensions become sufficiently small so that optical phonons are influenced by surface boundary conditions, a behavior well described by the dielectric continuum model for polar semiconductors [35].

The relative intensity and spectral width of the SM band are strongly influenced by the nanoparticle size and surface-to-volume ratio. In the sample synthesized at the lowest pulse energy (ZnSe_04), the SM band exhibits a pronounced intensity and definition, indicating a substantial contribution from surface vibrations. As the pulse energy increases, the relative intensity of the SM band decreases, and the LO mode becomes predominant. In the ZnSe_24 sample, additional low-frequency features appear at approximately 137 cm^−1^, 187 cm^−1^, and 202 cm^−1^, corresponding to the tranversal acoustic armonic (2TA), longitudinal acoustic (LA), and transvelsal optical (TO) phonon modes, respectively. The TO mode is also weakly evident as a shoulder in the ZnSe_18 sample, indicating an increased presence of bulk-like vibrational modes at higher pulse energies

**Figure 6:**
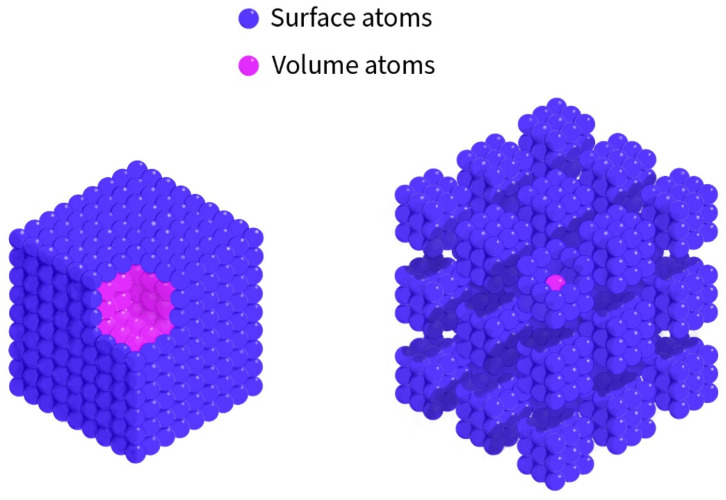
A schematic representation of the vibrational modes observed in ZnSe nanoparticles. The surface phonon mode (SM) arises from atoms located at or near the particle surface, while the longitudinal optical (LO) mode corresponds to vibrations within the crystalline volume.

A schematic representation of the LO and SM vibrational modes is shown in Figure 6, highlighting the physical origin of the surface phonon mode in polar nanocrystals.

**Figure 7:**
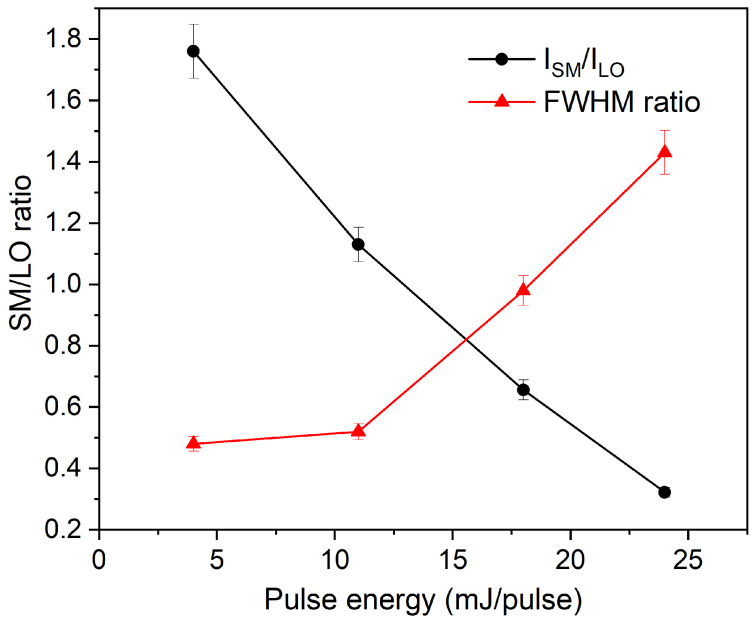
The variation in the SM/LO ratio as a function of pulse energy, determined using the area and the full width at half maximum (FWHM) of the peaks.

The evolution of the vibrational response was quantified by fitting the Raman spectra with Lorentzian functions for the SM and LO contributions. Integrated intensities (I) were determined from the fitted peak areas, and the ratio of the SM to LO intensity (I_SM_/I_LO_) was calculated for each sample (Figure 7). This ratio decreases monotonically with increasing pulse energies, ranging from 1.76 for ZnSe_04 to 0.32 for ZnSe_24. The full width at half maximum (FWHM) of the fitted SM and LO peaks was also analyzed. The FWHM ratio of the SM and LO modes increases nonlinearly with the pulse energy, which indicates a progressive broadening of surface-related vibrations relative to bulk-like modes. Specifically, in the ZnSe_04 sample, the LO mode is broader than the SM mode, suggesting a grater structural definition of the surface phonon contribution in this sample.

The change in the Raman response with pulse energy can be directly correlated with the size distributions observed via TEM. At a lower pulse energy, the higher fraction of small nanoparticles increases the surface-to-volume ratio, making surface atoms dominant in the vibrational response and enhancing the surface phonon mode. As the pulse energy increases, the contribution of larger, bulk-like fragments becomes more significant, reducing the relative intensity of the surface phonon mode and restoring the dominance of the longitudinal optical phonon.

The vibrational results are fully consistent with the optical results discussed in Section 3.3: the samples with the strongest SM component (ZnSe_04 and ZnSe_11) also display the largest blue shift in the absorption edge and the appearance of high-energy transitions in the derivative spectra. Together with the TEM observations and UV-Vis analysis, these results demonstrate that pulse energy is an effective parameter to tune the size, surface structure, and vibrational dynamics in ZnSe nanoparticles.

The simultaneous increase in I, the blue shift in the optical band gap, and the presence of a high-energy derivative feature in the absorption spectra all point toward a consistent size-dependent modification of the electronic and vibrational structure of ZnSe nanoparticles.

From a mechanistic perspective, these observations are consistent with a pulse-energy-dependent transition in the dominant fragmentation processes during LFL. This is confirmed by the systematic differences in particle sizes and properties revealed by the combined TEM, optical, and vibrational analyses. At lower pulse energies, fragmentation is likely governed predominantly by thermal processes, including surface heating, the partial evaporation of the parent particles, and the subsequent condensation of ablated species. This leads to the formation of nucleation centers and relatively small nanoparticles. In contrast, for samples synthesized at higher pulse energies, the fragmentation process appears to be dominated by more violent, non-thermal mechanisms. In this regime, spallation-like events may occur, associated with intense mechanical stress induced by cavitation bubble dynamics and/or shock waves generated upon laser–particle interactions. Such processes can result in the ejection of larger fragments, in agreement with the broader particle size distributions observed using TEM.

## 4. Conclusions

ZnSe nanoparticles were successfully synthesized via laser fragmentation in liquids at varying laser pulse energies, which enabled precise control over their structural, optical, and vibrational properties. Transmission electron microscopy, UV–Vis absorption, and Raman spectroscopy results demonstrate that the pulse energy determines the fragmentation regime during LFL and, consequently, the resulting nanoparticle size distribution. At lower pulse energies, TEM reveals a greater proportion of nanoparticles with sizes below the ZnSe exciton Bohr radius. This correlates with a pronounced blue shift in the optical band gap, the emergence of a high-energy feature in the derivative absorption spectra, and a dominant contribution of surface phonon modes, as indicated by the highest I_SM/I_LO ratios. Collectively, these findings indicate pronounced size- and surface-related effects associated with predominantly thermal fragmentation processes. Conversely, higher pulse energies produce broader size distributions that include larger, bulk-like fragments, resulting in a reduced relative contribution of surface vibrational modes and a smaller band gap shift, consistent with the activation of more disruptive, mechanically driven fragmentation mechanisms. XRD and SAED analyses indicate a predominance of the hexagonal phase in all samples and a preferential stabilization of hexagonal stacking sequences during laser fragmentation, with increasing structural disorder at higher pulse energies.

Overall, the regime-dependent behavior observed across all characterization techniques demonstrates that pulse energy serves as an effective parameter for tuning the nanoparticle size, the surface structure, and the coupled electronic and vibrational properties of ZnSe nanoparticles. These results underscore the potential of LFL as a surfactant-free method for producing II–VI semiconductor nanostructures with properties tailored for optoelectronic and photonic applications.

## Figures and Tables

**Table 1:** Laser pulse energy employed for the synthesis, and corresponding band gap and I_SM_/I_LO_ ratio of peak area and FWHM values obtained from Raman analysis for each sample.

Sample	Pulse Energy (mJ/pulse)	E_g_ (eV)	$I_{SM}/I_{LO}$	FWHM Ratio
ZnSe_04	4.4	2.83	1.76	0.48
ZnSe_11	11.2	2.77	1.13	0.52
ZnSe_18	18.4	2.77	0.66	0.98
ZnSe_24	24	2.71	0.32	1.43

**Table 2:** Measurements of SAED ring parameters and their associated families of crystalline planes.

Ring	r $\left[\frac{1}{nm}\right]$	d [nm]	Plane family	Ring	r $\left[\frac{1}{nm}\right]$	d [nm]	Plane family
ZnSe_04				ZnSe_24			
1	2.94	0.340	{100}	1	2.97	0.337	{100}
2	3.06	0.326	{002}	2	3.06	0.326	{002}
3	4.99	0.200	{110}	3	4.83	0.207	{110}
4	5.33	0.187	{103}	4	5.32	0.188	{103}
5	5.81	0.172	{112}	5	5.88	0.170	{112}

## Data Availability

The data presented in this study are available from the corresponding author upon reasonable request.

## Abbreviations

The following abbreviations are used in this manuscript:

LFL	Laser fragmentation in liquids
TEM	Transmission electron microscopy
LO	Longitudinal optic phonon
SM	Surface mode (phonon)
